# Equity in the Access of Chinese Immigrants to Healthcare Services in Portugal

**DOI:** 10.3390/ijerph20032442

**Published:** 2023-01-30

**Authors:** Sandra Lopes Aparício, Ivone Duarte, Luísa Castro, Rui Nunes

**Affiliations:** 1 Center for Health Technology and Services Research (CINTESIS), Faculty of Medicine, University of Porto, 4200-319 Porto, Portugal; 2 Department of Community Medicine, Information and Health Decision Sciences (MEDCIDS), Faculty of Medicine, University of Porto, 4200-319 Porto, Portugal; 3 School of Health of Polytechnic of Porto, 4200-072 Porto, Portugal

**Keywords:** healthcare access, health services access, Chinese, barriers, equity, traditional Chinese medicine (TCM)

## Abstract

International studies indicate that Chinese immigrants face barriers when trying to access healthcare in the host country. The aim of this study was to identify the barriers that Chinese immigrants face when accessing the Portuguese National Health Service. An observational, cross-sectional and quantitative study was carried out via a bilingual Portuguese/Mandarin self-completed paper questionnaire was applied. The study population consisted of individuals with Chinese nationality who were residing in mainland Portugal for at least one year and aged 18 years or over. A total of 304 individuals answered the questionnaire. The results show that 284 (93.4%) of the participants had already sought healthcare in Portugal. The participants identified language difficulties and health professionals’ lack of knowledge of Chinese cultural habits as the most significant barriers to accessing healthcare in Portugal. Of a total of 165 participants who sought healthcare in China, confidence in treatment outcomes and health professionals’ knowledge of Chinese cultural habits were the reasons given by 151 (91.5%) individuals. This study reveals the existence of linguistic and cultural barriers that can condition the access of the Chinese immigrant population to healthcare systems. Immigrants’ access to healthcare can be promoted via policies that contribute to proficiency in the Portuguese language and medical literacy among the Chinese immigrant population. It can also be promoted by raising the awareness of health professionals to Chinese cultural habits.

## 1. Introduction

Migration is common in the globalized world and is increasingly seen as a mandatory element for the economic and social development of countries [1]. The causes for migration are diverse. Some people move in search of new economic opportunities, while others move to escape armed conflict, poverty, food insecurity, persecution, terrorism or because of human rights violations [2]. According to the World Health Organization, there are currently one billion migrants of which 281 million are international migrants [3]. As a result of a favourable economic situation and an increase in the number of jobs, Portugal has seen an increase in the number of immigrants since 2015. The year 2020 was no exception with over 662,095 legalized immigrants registered corresponding to an increase of 12.2% versus 2019. Among the most representative foreign nationalities in Portugal, the Chinese represent the 7th largest community, with 26,074 citizens [4]. Despite the role that immigrants play in European societies, there are few studies in the scientific literature that focus on the health of immigrants. The under-representation of immigrants in health-related studies and the fact that surveys are not adapted to the context and language of immigrants may explain this observation. [5]. In the case of Chinese immigrants, beyond language barriers, they also often have long working hours and use Traditional Chinese Medicine (TCM) rather than Western healthcare [6]. Portugal faces the same reality where studies on the health of Chinese immigrants residing in the national territory are scarce [7]. The Chinese community is described as closed in on itself and rarely interacting with the indigenous population due to the fact of difficulties, not only linguistic but also cultural [8], which, according to the literature, leads them to be considered an “invisible minority” [9].

Newly arrived immigrants, regardless of their nationality, tend to be healthier than the general population. However, over time, the health of immigrants declines and approaches the state of health of the native population, which can be described as the “healthy immigrant effect” [10,11,12]. Several factors can contribute to the decline in the health status of immigrants: discrimination, language, cultural barriers, and difficulties in accessing health services [12,13]. This decline may also result from the process of the acculturation of immigrants to the new culture by adopting the behaviours, attitudes, beliefs, and language of the indigenous population [11,13,14]. There is no consensus on the pattern of use of health services by immigrants. Some studies indicate that immigrants often use healthcare, while others report no significant effect. However, there is consensus on the overuse of emergency services by immigrants. Ease of access is one commonly justified reasons [15].

The Constitution of the Portuguese Republic guarantees access to healthcare for all, including foreign citizens [16]. Although Chinese immigrants are allowed access to the Portuguese National Health Service (SNS), their visibility as users of the SNS is small when compared to other immigrant communities [17]. International studies indicate that Chinese immigrants seek health services on a smaller scale than other immigrant communities [7,9]. The use of TCM [18,19,20,21], self-medication [22], the search for healthcare in China [10,23], the language barrier and the time spent [24] could be factors that explain the behaviour of these immigrants [25].

The United Nations considers health to be a fundamental right of human beings, without restrictions due to the fact of race, colour, sex, language, religion, political opinions, nationality or social origin [26]. Faced with the right to health, governments are impelled to promote policies that enable the population to be as healthy as possible [27].

The literature states that Chinese immigrants, like other ethnic minorities, have great difficulties when trying to access healthcare in the host country [25]. Thus, it is important to ascertain whether the right to health, justice and equity [28] are ensured in the access of Chinese immigrants, residing in mainland Portugal, to healthcare provided by the SNS.

The main objective of this study was to identify the barriers that the Chinese immigrant population faces in accessing the Portuguese SNS and the associated factors that limit this population’s access to healthcare.

## 2. Materials and Methods

### 2.1. Study Design

This was an observational, cross-sectional and quantitative study. The questionnaire used here was built based on the existing literature, in a bilingual Portuguese/Mandarin version, with the instrument being translated and back-translated to guarantee equivalence of the versions. Mandarin was used because it is the standard dialect of the Chinese language. The participants were individuals with Chinese nationality, residing in mainland Portugal for at least one year and aged 18 years or over.

### 2.2. Instrument

The questionnaire was divided into three sections.

#### 2.2.1. Section I—Socio-Professional Characterization

The variables under study were sex (female/male), age in years, place of origin (Mainland China/Hong Kong/Macau/other), place of residence (North/Algarve/Alentejo/Centre/Lisbon Metropolitan Area), civil status (single/married/divorced or separated/widowed), length of residence in Portugal (from 1 to 5 years/from 6 to 10 years/more than 11 years), knowledge of languages (Portuguese/English/French/Spanish/others), level of education (basic education/secondary education/higher education) and professional sector (trade/catering/real estate/teaching—translation/import—export/others).

#### 2.2.2. Section II—Access to Healthcare in Portugal

The variables under study were enrolment in the Portuguese SNS (yes/no), healthcare providers (health centre/physiotherapy clinic/clinic—private hospital/dental care clinic/pharmacy/public hospital/INEM/SNS 24 contact/emergency service) and nationality of doctors (Chinese/other nationality), reasons for seeking healthcare (car accident/personal accident/accident at work/chronic illness monitoring/pregnancy monitoring/preventive healthcare/childbirth/child health/vaccination/dentistry/surgical treatment/medical treatment/radiotherapy/chemotherapy/cold–flu/diarrhoea/vomiting/chronic illness/diabetes/heart disease/oncological disease/liver disease/respiratory disease/neurological disease/psychiatric disease/musculoskeletal disease/stomach–intestinal disease/clinical analysis/ultrasound/CAT scan/NMR/X-ray/PET scan/other) and barriers in accessing healthcare (language/lack of knowledge of Chinese habits/hours/price/administrative barriers/prejudice towards Western healthcare/lack of knowledge of how it works).

#### 2.2.3. Section III—Looking for Healthcare in China

The variables under study were demand for healthcare in China (yes/no), type of illness that motivated the search for healthcare in China (chronic disease monitoring/preventive care/stomach–intestinal disease/musculoskeletal disease/medical treatment/respiratory disease/surgical treatment/accident at work/liver disease/heart disease/psychiatric disease/pregnancy monitoring/diabetes/oncological disease/vaccination/neurological disease/personal accident/childbirth/child health/chemotherapy/radiotherapy/dentistry/car accident/others), reasons for seeking healthcare in China (confidence in the outcome of treatments/knowledge on the part of health professionals of the cultural habits of the Chinese population/family support/health professionals of Chinese origin/experience prior notice from himself or other community members/cost/others), shipment of TCM medication to Portugal (yes/no), type of illness that motivated the request for TCM medication (pregnancy monitoring/preventive healthcare/childbirth/child health/vaccination/cold–flu/diarrhoea/vomiting/diabetes/heart disease/oncological disease/liver disease/respiratory disease/neurological disease/psychiatric disease/musculoskeletal disease/stomach–intestinal disease) and reasons for requesting TCM medication (confidence in the outcome of treatments/health professionals’ knowledge of TCM cultural habits Chinese population/family support/health professionals of Chinese origin/previous experience of own or other members of the community/cost/others).

### 2.3. Procedures

Data collection took place from January to June 2019. The data collection instrument was self-completed on paper. Before being applied within the Chinese community, a pre-test of the questionnaire was performed via a convenience sample composed of ten Chinese with different qualifications and activities. The questions were considered clear and understood by the participants. This allowed the questionnaire to be applied to the target population. The questionnaires were administered by a member of the bilingual Chinese community who had been trained prior to data collection; the medical terminology used in the questionnaire was explained to him. Despite the questionnaire being filled out individually, the researcher and the member of the bilingual community were available to help with completing it and clarifying questions that raised doubts for the respondents.

The respondents were mainly recruited at their place of work, in Chinese shops, supermarkets, and Chinese restaurants. Passers-by were also approached in areas known as Chinatown (both in the north and south), as well as at the Chinese School, located in the city of Lisbon. The participants were asked to respond to the questionnaire individually.

To ensure a representative sample of the Chinese immigrant population, an attempt was made to match men and women in equal numbers [29]. Approximately 65% of respondents were willing to collaborate; however, people from older age groups showed a greater reluctance to answer the questionnaire, as did males. The response time varied because the questionnaires were usually answered at the respondents’ workplace and during office hours. Data were collected anonymously, after a brief explanation of the scope and purpose of the study in Mandarin. All respondents signed a consent form to participate in the study.

### 2.4. Statistical Method

The data obtained were entered and analysed in the SPSS software (software package SPSS, version 26.0 for Windows IBM SPSS Inc. (Chicago, IL, USA)). The categorical variables were described by absolute and relative frequencies, *n* (%). The quantitative variable age, as it presented an asymmetrical distribution, was described by the median and interquartile range, Med [Q1, Q3]. The association between categorical variables was analysed using the Chi-square test, and the comparison of the distribution of the age variable between two groups was performed using the nonparametric Mann–Whitney test. *p*-Values < 0.05 were considered significant in all statistical tests.

## 3. Results

### 3.1. Sample Characteristics

The sample characteristics are described in Table 1. Of the 304 participants, 153 (50.3%) are men. The sample is equally distributed between men and women, in accordance with official SEF data for the year 2020 [29]. When asked, most participants (*n* = 293; 96.4%) say they speak Portuguese. In line with the data collection location, 264 (86.8%) of the participants reported working in stores and 25 (8.2%) in restaurants.

### 3.2. Search for Healthcare in Portugal

Of the 304 participants, 285 (93.8%) are enrolled in the Portuguese National Health Service (SNS). Most participants, 284 (93.4%), answered that they had already needed healthcare in Portugal. Western medicine was the choice of 283 (99.6%) of the participants, with 69 (24.3%) referring to the use of at least one CTM therapy. The participants resorted mainly to health centres (*n* = 265; 93.3%), pharmacies (*n* = 247; 87%) and public hospitals (*n* = 236; 83.1%). For the most part, the health professionals who provided healthcare to the participants were not of Chinese ethnicity (see Table S1 in the Supplementary Materials). Table S2 (Supplementary Materials) lists the reasons that led Chinese immigrants to seek healthcare. It might be possible that the immigrants had resorted to CTM and/or Western medicine within which the split into public and private sectors was performed. The demand for healthcare in CTM was residual versus Western medicine.

### 3.3. Barriers to Accessing Healthcare

The study participants listed some of the barriers they faced in accessing healthcare. Communication difficulties and lack of knowledge on the part of health professional, including the cultural habits of the Chinese population are presented as barriers to accessing healthcare. They were mentioned by 157 (51.6%) and 129 (42.4%) of the participants, respectively. In the public sector, 102 (33.6%) of the participants considered appointment time another element that conditions access to the SNS. The barriers are detailed in Table 2 based on sector and type of medicine.

The data indicate that age (*p* = 0.007), sex (*p* = 0.006) and length of residence in Portugal (*p* = 0.010) were significant variables in identifying language as a barrier to accessing healthcare in Portugal. In particular, the participants who indicated language as a barrier tended to be older (median 40 vs. 38 years), male (59.6% vs. 47.7% of women) and have lived in Portugal for less time (see Table 3).

It appears that the Chinese population who completed secondary education in China is more likely to mention professionals’ lack of knowledge of Chinese habits as a barrier to accessing healthcare in Portugal (see Table 4).

### 3.4. Searching for Healthcare in China

China was chosen by 165 (54.3%) of the participants to receive health care. The monitoring of chronic diseases as well as the demand for preventive healthcare, musculoskeletal diseases and stomach diseases were some of the health problems that justified the demand for healthcare in China (see Table S3 in Supplementary Materials).

Knowledge on the part of health professionals regarding Chinese cultural habits and confidence in the outcome of treatments were reported by 151 (91.5%) of the participants who resorted to healthcare in China. Family support and the nationality of health professionals were other reasons cited (see Table 5).

The data indicate that the level of education (*p* < 0.001), age (*p* < 0.001) and length of residence in Portugal (*p* < 0.001) are variables significantly associated with the search for healthcare in China. In particular, participants seeking healthcare in China are older (median 43 vs. 30 years), have lower education and have lived longer in Portugal (see Table 6). 

### 3.5. Shipment of TCM Medication

Most of the population that participated in the study (*n* = 225; 74%) answered that they have asked family and friends to send them TCM medicines from China to fight the cold/flu [183 (81.3%)], intestinal tract problems [65 (28.9%)] and stomach/bowel diseases [62 (27.6%)]. This medication is used to prevent health problems by 157 (69.8%) of the 225 participants who requested the medication to be sent. Confidence and previous experience—either by oneself or by other members of the community—in the use of medicines were the reasons invoked by 214 (95.1%) and 120 (53.3%), respectively, of the study participants. Cost does not play a relevant role in decision making and was mentioned by a small number of Chinese immigrants, i.e., only 7 (3.1%) of the 225 participants. In terms of ordering medicines from China, no significant associations were found with sociodemographic variables (Table 7).

## 4. Discussion

This study demonstrates that most participants resort to healthcare from the Portuguese SNS. The barriers that Chinese immigrants face do not prevent their search for healthcare. This result differs from studies carried out in other countries [30]. The results obtained seem to corroborate the existing scientific literature by confirming that language is a barrier to accessing the Portuguese SNS [5,9,24], although the vast majority of respondents answered that they speak Portuguese, which leads us to question whether the problem stems from the use of medical terminology by health professionals [31] or whether this group simply has a limited command of the Portuguese language [9]. It should be noted that language barriers are associated with inequities in access to healthcare and in the clinical results obtained. Several studies report that patients who speak the local language have more satisfactory clinical results than those who face language barriers [31]. Language barriers can even lead to clinical errors by preventing appropriate communication between healthcare providers and patients [32].

Our results illustrate that study participants noted a lack of knowledge of the cultural habits of the Chinese population on the part of health professionals as a barrier in accessing the SNS. This lack of knowledge is not mentioned by health professionals, but the perception that the Chinese have regarding the lack of knowledge of the cultural habits of the Chinese population by health professionals. However, it is important to note that health professionals in Portugal do not receive specific training in the area of multiculturalism, which can condition the type of healthcare provided to immigrant populations [33]. However, this situation might dramatically change in the coming years because the Portuguese universal educational system has evolved in the last decade to include mandatory education for values and human rights in a multicultural perspective. Indeed, the mandatory discipline in elementary school of Education for Citizenship includes education for human rights, nondiscrimination, and inclusion of all people [34]. In the future, education for digital inclusion will likely follow the same path [35]. 

The participants in our study considered hours for outpatient consultations at hospitals in the SNS network to be a barrier. This fact is not unrelated related to the long hours that Chinese immigrants often work. Indeed, the right to healthcare access of appropriate quality [36] implies full accessibility to health services. It follows that the reorganization of the services should be implemented so that good care is delivered considering the health needs of all people, regardless of their origin or level of income. Only in this way is the right to healthcare access of appropriate quality more than an aspirational desire. Most of the time, the remuneration earned may be related to the number of hours worked and, thus, scheduling appointments during working hours may imply a reduction of income [22].

Several studies report that immigrants consider that accessing and using health services is an obstacle along with linguistic and cultural barriers [27,37]. However, our results do not reflect this reality. Chinese immigrants residing in Portugal do not consider lack of knowledge regarding the functioning of health services and the existing administrative barriers as conditioning factors in accessing healthcare contrary to what happens in other countries [31,38].

Another important result is that the process of acculturation of the Chinese immigrant population—as demonstrated by the high rate of participants who admit to resorting to Western medicine—does not necessarily mean abandoning their beliefs in relation to TCM [9]. As described in other scientific studies, the participants in our study reported resorting to both Western medicine and the use of TCM medicines to meet their healthcare needs [10,22]. In general, the Chinese population accepts the complementarity of TCM with Western medicine. They perceive that the synergies generated by the two medicines favour a satisfactory clinical result [39].

Additionally, and in line with what occurs with Chinese immigrant communities living in other countries, a significant number of participants admitted to having travelled to China to access the Chinese health system. The search for healthcare in the country of origin seems to indicate that immigrants’ resort to health services perceived as more culturally and linguistically appropriate [5,10,24].

## 5. Conclusions

These results demonstrate that the study participants resort, for the most part, to healthcare provided in the Portuguese SNS. However, there is still a need to adopt health policies that enable greater equity in access to healthcare by this community. 

It seems necessary to implement projects that promote proficiency in the Portuguese language and medical literacy among Chinese immigrants, as well as raising the awareness of health professionals to Chinese cultural habits and the use of TCM by the Chinese immigrant community. The offer of Mandarin courses for health professionals should be considered. The hiring of health professionals with proficiency in Mandarin and/or Chinese nationality should also be evaluated. These two measures would facilitate communication and understanding between health professionals and Chinese immigrants, with a direct impact on the quality of services provided. The creation of a support office composed of employees/technicians with knowledge of Mandarin and/or Chinese culture as well as the preparation of assistance guides and dossiers with the most important and relevant characteristics of Chinese culture could also be implemented. Better articulation with the SNS should be studied, and the respective impact within the Chinese community should be assessed.

Portugal is recognized for promoting integration policies with immigrant communities and for welcoming these same communities into Portuguese society. The adoption of measures that facilitate immigrants’ access to healthcare would provide a better integration of these communities and improve health indicators, while guaranteeing that the right to health, justice, and equity are assured for this population.

### Study Limitations

This study does have some limitations. The fact that a convenience sample was used makes it difficult to infer that our sample, despite having a reasonable size, is representative of the Chinese community residing in mainland Portugal. The respondents were recruited mainly from Chinese stores and restaurants, which deprived us of members of the community whose professional activity requires higher levels of education (university students and researchers) as well as individuals without a professional occupation (unemployed and retired) and depending on the age group of the respondents.

## Figures and Tables

**Table 1:** Characteristics of the participants (*n* = 304).

Variable	*n*	%
**Sex**		
Male	153	50.3
Female	151	49.7
**Age (years), med [Q1; Q3]**	40 [30; 48]	
**Origin**		
Mainland China	300	98.7
Macau	3	1.0
Hong Kong	1	0.3
**Civil status**		
Unmarried	63	20.7
Married	228	75.0
Divorced or separated	8	2.6
Windowed	5	1.6
**Level of education**		
Basic education	170	55.9
Secondary education	102	33.6
Higher education	32	10.5
**Foreign languages**		
Portuguese	293	96.4
English	58	19.1
French	3	1
Spanish	9	3
Others	9	3
**Residence (NUTS II)**		
North	66	21.7
Algarve	27	8.9
Centre	56	18.4
Lisbon Metropolitan Area	124	40.8
Alentejo	31	10.2
**Years in Portugal**		
From 1 year to 5 years	53	17.4
From 6 years to 10 years	74	24.3
More than 11 years	177	58.2
**Professional field** *		
Shops	264	86.8
Restaurant	25	8.2
Real estate	5	1.6
Teaching/translation	3	1
Import/export	8	2.6
Others	3	1

* Each participant could choose more than one option (hence, the sum being > 304).

**Table 2:** Barriers to accessing healthcare in Portugal pointed out by Chinese immigrants (*n* = 304).

	Western Medicine Private Sector, *n* (%)	Western Medicine/Public/Private Sectors, *n* (%)	Western Medicine Public Sector, *n* (%)	TCM, *n* (%)	TCM/Western Medicine Private Sector, *n* (%)	TCM/Western Medicine Public Sector, *n* (%)	None, *n* (%)
Idiom	0 (0)	11 (3.6)	145 (47.7)	1 (0.3)	0 (0)	0 (0)	147 (48.4)
Unfamiliarity Chinese habits	0 (0)	15 (4.9)	111 (36.5)	0 (0)	0 (0)	3 (1)	175 (57.6)
Schedule	3 (1)	3 (1)	102 (33.6)	0 (0)	0 (0)	0 (0)	196 (64.5)
Price	57 (18.8)	2 (0.7)	3 (1)	14 (4.6)	4 (1.3)	1 (0.3)	223 (73.4)
Administrative barriers	0 (0)	1 (0.3)	58 (19.1)	0 (0)	0 (0)	0 (0)	245 (80.6)
Western healthcare bias	0 (0)	9 (3)	47 (15.5)	0 (0)	0 (0)	0 (0)	248 (81.6)
Unfamiliarity with the operations	0 (0)	1 (0.3)	28 (9.2)	0 (0)	0 (0)	0 (0)	275 (90.5)

Each participant could choose more than one option (hence, the sum being >304).

**Table 3:** Comparison of the variables sex, age, length of residence in Portugal, level of education and where you attended secondary school according to the indication of language as a barrier to accessing healthcare in Portugal (*n* = 304).

	Idiom Not a Barrier (*n* = 147)	Idiom as a Barrier (*n* = 157)	*p*-Value
Sex, *n* (%)			0.006 ^a^
Male	61 (40.4%)	90 (59.6%)	
Female	79 (52.3%)	72 (47.7%)	
Age, med [1Q; 3Q]	38 [25; 46]	40 [31; 49.5]	0.007 ^b^
Length of residence in Portugal, *n* (%)			0.010 ^a^
Between 1 and 5 years	23 (43.4%)	30 (56.6%)	
Between 6 and 10 years	26 (35.1%)	48 (64.9%)	
Over 10 years	98 (55.4%)	79 (44.6%)	
Level of education, *n* (%)			0.222 ^a^
Basic	75 (44.1%)	95 (55.9%)	
Secondary	56 (54.9%)	46 (45.1%)	
Higher	16 (50.0%)	16 (50.0%)	
Where you attended secondary school, *n* (%)			<0.001 ^a^
China	116 (43.4%)	151 (56.6%)	
Other country	31 (83.8%)	6 (16.2%)	

^a^ Chi-square test; ^b^ Mann–Whitney test.

**Table 4:** Comparison of the variables sex, age, length of residence in Portugal, level of education and where you attended secondary school according to the indication of lack of knowledge of Chinese habits as a barrier to accessing healthcare in Portugal (*n* = 304).

	Chinese Habits Not a Barrier (*n* = 175)	Chinese Habits as a Barrier (*n* = 129)	*p*-Value
Sex, *n* (%)			0.066 ^a^
Male	96 (62.7%)	57 (37.3%)	
Female	79 (52.3%)	72 (47.7%)	
Age, med [1Q; 3Q]	38 [28; 47]	40 [30; 49]	0.134 ^b^
Length of residence in Portugal, *n* (%)			0.626 ^a^
Between 1 and 5 years	33 (62.3%)	20 (37.7%)	
Between 6 and 10 years	44 (59.5%)	30 (40.5%)	
Over 10 years	98 (55.4%)	79 (44.6%)	
Level of education, *n* (%)			0.985 ^a^
Basic	97 (57.1%)	73 (42.9%)	
Secondary	59 (57.8%)	43 (42.2%)	
Higher	19 (59.4%)	13 (40.6%)	
where you attended secondary school, *n* (%)			0.017 ^a^
China	147 (55.1%)	120 (44.9%)	
Other country	28 (75.7%)	9 (24.3%)	

^a^ Chi-square test; ^b^ Mann–Whitney test.

**Table 5:** Reasons for seeking healthcare in China (*n* = 165).

Reasons	*n*	%
Confidence in treatment results	151	91.5
Knowledge by health professionals of the cultural habits of the Chinese population	151	91.5
Family support	119	72.1
Chinese health professionals	118	71.5
Previous experience of own or other members of the community	90	54.5
Other	7	4.2
Cost	6	3.6

Each participant could choose more than one option (hence, the sum being >165).

**Table 6:** Comparison of the variables sex, age, length of residence in Portugal and level of education according to the demand for healthcare in China (*n* = 304).

	Does Not Seek Healthcare in China (*n* = 139)	Seeks Healthcare in China (*n* = 165)	*p*-Value
Sex, *n* (%)			0.638 ^a^
Masculine	72 (47.1%)	81 (52.9%)	
Feminine	67 (44.4%)	84 (55.6%)	
Age, med [1Q; 3Q]	30 [25; 42]	43 [35; 51.5]	<0.001 ^b^
Length of residence in Portugal, *n* (%)			<0.001 ^a^
Between 1 and 5 years	37 (69.8%)	16 (30.2%)	
Between 6 and 10 years	41 (55.4%)	33 (44.6%)	
Over 10 years	61 (45.7%)	116 (65.5%)	
Level of education, *n* (%)			<0.001 ^a^
Basic	55 (32.4%)	115 (67.6%)	
Secondary	60 (58.8%)	42 (41.2%)	
Higher	24 (75%)	8 (25%)	

^a^ Chi-square test; ^b^ Mann–Whitney test.

**Table 7:** Comparison of the variables sex, age, length of residence in Portugal and level of education according to the ordering of medications from China (*n* = 304).

	Does Not Order Medication from China (*n* = 79)	Orders Medication from China (*n* = 225)	*p*-Value
Sex, *n* (%)			0.746 ^a^
Male	41 (26.8%)	112 (73.2%)	
Female	38 (25.2%)	113 (74.8%)	
Age, med [1Q; 3Q]	36 [26; 47]	40 [30; 49]	0.071 ^b^
Length of residence in Portugal, *n* (%)			0.434 ^a^
Between 1 and 5 years	15 (28.3%)	38 (71.7%)	
Between 6 and 10 years	15 (20.3%)	59 (79.7%)	
Over 10 years	49 (27.7%)	128 (72.3%)	
Level of education, *n* (%)			0.128 ^a^
Basic	40 (23.5%)	130 (76.5%)	
Secondary	26 (25.5%)	76 (74.5%)	
Higher	13 (40.6%)	19 (59.4%)	

^a^ Chi-square test; ^b^ Mann–Whitney test.

## Data Availability

The exact data can be obtained from the corresponding author.

## Supplementary Materials

The following are available at: https://www.mdpi.com/article/10.3390/ijerph20032442/s1, Table S1: Healthcare providers used by the Chinese population residing in mainland Portugal. Table S2: Health reasons for seeking healthcare in Portugal. Table S3. Health reasons for seeking healthcare in China.

## References

[1] Davies A., Basten A., Frattini C. (2009). Migration: A Social Determinant of the Health of Migrants. Eurohealth.

[2] United Nations (2016). Res 71/1 New York Declaration for Refugees and Migrants (3 October 2016). Volume 16163. https://academic.oup.com/ijrl/article-lookup/doi/10.1093/ijrl/eew057.

[3] World Health Organization Refugee and Migrant Health. https://www.who.int/health-topics/refugee-and-migrant-health#tab=tab_1.

[4] Reis S., Sousa P., Machado R. Relatório de Imigração, Fronteiras e Asilo 2020. Serviço de Estrangeiros e Fronteiras. Published 2021. http://sefstat.sef.pt/Docs/Rifa_2020.pdf.

[5] Badanta-Romero B., Lucchetti G., Barrientos-Trigo S. (2021). Access to healthcare among Chinese immigrants living in Seville, Spain. Gac. Sanit..

[6] Badanta B., Rodríguez-Burbano A.Y., López-Tarrida A.C., Vega-Escaño J., Lucchetti G., Barrientos-Trigo S., de Diego-Cordero R. (2021). Health Problems and the Use of Medications and Traditional Therapies among Chinese Immigrants Living in Spain. Healthcare.

[7] Fernandes A.I.P. Trânsitos Terapêuticos—Uma Biografia entre Lisboa e Zhejiang. Tese Mestr Univ Nov.:3. https://run.unl.pt/handle/10362/20778.

[8] Gonçalves C.M.D. (2018). Na Cabeça Do Dragão.

[9] Liu C.H., Ingleby D., Meeuwesen L. (2011). Barriers to Health Care for Chinese in the Netherlands. Int. J. Fam. Med..

[10] Ou C.H.K., Wong S.T., Levesque J.F., Saewyc E. (2017). Healthcare needs and access in a sample of Chinese young adults in Vancouver, British Columbia: A qualitative analysis. Int. J. Nurs. Sci..

[11] Pandey M., Maina R.G., Amoyaw J., Li Y., Kamrul R., Michaels C.R., Maroof R. (2021). Impacts of English language proficiency on healthcare access, use, and outcomes among immigrants: A qualitative study. BMC Health Serv. Res..

[12] Karmali N. (2021). The Healthy Immigrant Effect: Is Canada’s Health System Failing Immigrants?. Glob. Health Ann. Rev..

[13] Moniz A.M.F. (2019). Healthy Immigrant Effect Em Portugal: Estudo Sobre Os Imigrantes Extra UE Presentes No Inquérito Nacional de Saúde 2014. Ph.D. Thesis.

[14] Kan K.L., Connor H., Beddoe L. (2020). Accessing social service support: Barriers experienced by Chinese migrants living in Auckland, Aotearoa New Zealand. Aotearoa New Zeal. Soc. Work..

[15] Coutinho G.N.S., da Cunha P. Acesso aos Cuidados de Saúde das Comunidades Imigrantes em Portugal. 2016. (Query date: 2021-03-18 14:16:41). https://repositorio.ul.pt/handle/10451/26721.

[16] Almedina (2021). Constituição da República Portuguesa.

[17] Pereira M.A.D.A. (2006). A Comunidade Chinesa Imigrante em Portugal e os Cuidados de Saúde: Um Estudo na Região de Lisboa.

[18] Tong M., Sentell T. (2017). Insights in Public Health: Challenges Investigating Health Outcomes in Chinese Americans Using Population-Based Survey Data. Hawaii J. Med. Public Health.

[19] Yang L.H., Corsini-Munt S., Link B.G., Phelan J.C. (2009). Beliefs in traditional chinese medicine efficacy among chinese americans: Implications for mental health service utilization. J. Nerv. Ment. Dis..

[20] Wu A.P.W., Burke A., LeBaron S. (2007). Use of traditional medicine by immigrant Chinese patients. Fam. Med..

[21] Kong Y., Shaver L.G., Shi F., Yang L., Zhang W., Wei X., Zhu Y., Wang Y., Wang P.P. (2021). Attitudes of Chinese immigrants in Canada towards the use of Traditional Chinese Medicine for prevention and management of COVID-19: A cross-sectional survey during the early stages of the pandemic. BMJ Open.

[22] Tam W.J., Goh W.L., Chua J., Legido-Quigley H. (2017). Health is my capital: A qualitative study of access to healthcare by Chinese migrants in Singapore. Int. J. Equity Health.

[23] Qiu J., Song D., Nie J., Su M., Hao C., Gu J., Hao Y., Kiarie J.N., Chung M.H. (2019). Utilization of healthcare services among Chinese migrants in Kenya: A qualitative study. BMC Health Serv. Res..

[24] Sarpel U., Huang X., Austin C., Gany F. (2018). Barriers to Care in Chinese Immigrants with Hepatocellular Carcinoma: A Focus Group Study in New York City. J. Community Health.

[25] Kwok H.H.Y., Low J., Devakumar D., Candy B. (2020). Experience and perspectives on palliative or end-of-life care of Chinese people and their families as immigrants to high-income countries: A systematic review and thematic synthesis. BMJ Glob. Health.

[26] Neal A.C. (2019). United Nations Universal Declaration of Human Rights (1948) 1. Fundamental Social Rights at Work in the European Community.

[27] Devillé W., Lamkaddem M. (2011). Project Report on Delphi Process on Best Practice of Health Care for Immigrants. http://hdl.handle.net/2078.1/74645.

[28] (2004). ACC Aristóteles—Ética a Nicómaco.

[29] Serviço de Estrangeiros e Fronteiras SEFSTAT—Portal de Estatística do, S.E.F. População Estrangeira Residente em Portugal. Published 2022. https://sefstat.sef.pt/forms/distritos.aspx.

[30] Al Shamsi H., Almutairi A.G., Al Mashrafi S., Al Kalbani T. (2020). Implications of language barriers for healthcare: A systematic review. Oman Med. J..

[31] Appoh L., Felix F., Pedersen P.U. (2020). Barriers to access of healthcare services by the immigrant population in Scandinavia: A scoping review protocol. BMJ Open.

[32] Cohen A.L., Rivara F., Marcuse E.K., McPhillips H., Davis R. (2005). Are language barriers associated with serious medical events in hospitalized pediatric patients?. Pediatrics.

[33] Estrela P. (2009). A saúde dos imigrantes em Portugal. Rev. Port. Clínica Geral..

[34] Nunes R., Duarte I., Santos C., Rego G. (2015). Education for values and bioethics. Springerplus.

[35] Kooli C., Al Muftah H. (2022). Artificial intelligence in healthcare: A comprehensive review of its ethical concerns. Technol. Sustain..

[36] Nunes R. (2022). Healthcare as a Universal Human Right: Sustainability in Global Health.

[37] Hyatt A., Lipson-Smith R., Schofield P., Gough K., Sze M., Aldridge L., Goldstein D., Jefford M., Bell M.L., Butow P. (2017). Communication challenges experienced by migrants with cancer: A comparison of migrant and English-speaking Australian-born cancer patients. Health Expect..

[38] Adhikari M., Kaphle S., Dhakal Y., Duwadi S., Subedi R., Shakya S., Tamang S., Khadka M. (2021). Too long to wait: South Asian migrants’ experiences of accessing health care in Australia. BMC Public Health.

[39] Chung V.C., Lau C.H., Yeoh E.K., Griffiths S.M. (2009). Age, chronic non-communicable disease and choice of traditional Chinese and western medicine outpatient services in a Chinese population. BMC Health Serv. Res..

