# Supplementary material for: Equity in the Access of Chinese Immigrants to Healthcare Services in Portugal
Source: Int J Environ Res Public Health. 2023 Jan 30;20(3):2442. doi: 10.3390/ijerph20032442 (PMC9916350; doi:10.3390/ijerph20032442)
Supplement: Supplementary file 1 [file ijerph-20-02442-s001.zip › ijerph-2144640-supplementary.pdf]

Table S1. Healthcare providers used by the Chinese population residing in mainland Portugal (n = 284).

Healthcare Services Used	None		Chinese Doctor		Doctor of Another Nationality	
	n	%	n	%	n	%
Health Centre	19	6.7	19	6.7	246	86.6
Physiotherapy's clinic	265	93.3	3	1.1	16	5.69
Private Clinic / Hospital	198	69.7	19	6.4	68	23.9
Dental Care Clinic	192	67.6	5	1.8	87	30.6
Pharmacy	37	13	19	6.7	228	80.3
Public hospital	48	16.9	22	7.7	214	75.4
INEM	268	94.4	2	0.7	14	4.9
Healthcare 24h (online service 24/7)	274	96.5	0	0	10	3.5
Emergency Service	201	70.8	5	1.6	78	25.7

Each participant could choose more than one option (hence the sum being >284).

Table S2. Health reasons for seeking healthcare in Portugal.

Reasons (n=304)	Western Medicine (Public and Private Sectors)	TCM	TCM/Western Medicine (Public and Private Sectors)	None	All
	n (%)	n (%)	n (%)	n (%)	n (%)
Car Accident	10 (3.5)	0 (0)	0 (0)	274 (96.5)	0 (0)
Personal Accident	21 (7.4)	1 (0.4)	5 (1.8)	256 (90.1)	1 (0.4)
Work Accident	20 (7.1)	1 (0.4)	1 (0.4)	261 (91.9)	1 (0.4)
Chronic Disease Monitoring	227 (79.9)	1 (0.4)	14 (4.9)	42 (14.8)	0 (0)
Pregnancy Monitoring	68 (24)	0 (0)	4 (1.4)	212 (74.6)	0 (0)
Preventive Care	74 (26.1)	6 (2.1)	18 (6.3)	186 (65.5)	0 (0)
Childbirth	71 (25)	0 (0)	0 (0)	213 (75)	0 (0)
Children's health	78 (27.5)	0 (0)	0 (0)	206 (72.5)	0 (0)
Vaccination	70 (24.7)	0 (0)	0 (0)	214 (75.4)	0 (0)
Dentistry	57 (20,1)	0 (0)	0 (0)	227 (79.9)	0 (0)
Surgical treatment	30 (10.6)	0 (0)	0 (0)	254 (89.4)	0 (0)
Medical treatment	46 (16.2)	0 (0)	0 (0)	238 (83.8)	0 (0)
Radiotherapy	4 (1.5)	0 (0)	0 (0)	280 (98.6)	0 (0)
Chemotherapy	3 (1.1)	0 (0)	0 (0)	281 (98.9)	0 (0)
Cold - Flu	137 (48.2)	4 (1.4)	9 (3.2)	134 (47.2)	0 (0)
Diarrhoea	35 (12.3)	1 (0.4)	4 (1.4)	244 (85.9)	0 (0)
Vomit	34 (12)	1 (0.4)	3 (1.1)	246 (86.6)	0 (0)
Diabetes	14 (4.9)	0 (0)	1 (0.4)	269 (94.7)	0 (0)
Heart disease	10 (3.5)	0 (0)	1 (0.4)	273 (96.1)	0 (0)
Oncological Disease	4 (1.5)	0 (0)	2 (0.7)	277 (97.5)	1 (0.4)
Liver disease	10 (3.5)	1 (0.4)	0 (0)	273 (96.1)	0 (0)
Respiratory disease	24 (8.5)	3 (1.1)	6 (2.1)	251 (88.4)	0 (0)
Neurological Disease	6 (2.1)	0 (0)	2 (0.7)	276 (97.2)	0 (0)
Psychiatric Disease	12 (4.3)	1 (0.4)	1 (0.4)	270 (95.1)	0 (0)
Musculoskeletal Disease	19 (6.7)	0 (0)	5 (1.8)	258 (90.8)	2 (0.7)
Stomach/Intestine Disease	48 (16.9)	1 (0.4)	9 (3.2)	225 (79.2)	1 (0.4)
Other	8 (2.8)	0 (0)	0 (0)	276 (97.2)	0 (0)
Clinical analysis	274 (96.5)	0 (0)	1 (0.4)	9 (3.2)	0 (0)
Ultrasound	174 (61.2)	0 (0)	0 (0)	110 (38.7)	0 (0)
CAT scan	109 (38.4)	0 (0)	0 (0)	175 (61.6)	0 (0)
NMR	26 (9.2)	0 (0)	0 (0)	258 (90.8)	0 (0)
X-ray	81 (28.5)	0 (0)	0 (0)	203 (71.5)	0 (0)
PET scan	9 (3.2)	0 (0)	0 (0)	275 (96.8)	0 (0)
Other	38 (13.4)	0 (0)	0 (0)	246 (86.6)	0 (0)

Table S3. Health reasons for seeking healthcare in China (n=165).

Health Problem	n	%
Chronic Disease Monitoring	140	84.8
Preventive Care	63	38.2
Stomach-Intestine Disease	42	25.5
Musculoskeletal Disease	26	15.8
Medical treatment	17	10.3
Respiratory disease	16	9.7
Surgical treatment	13	7.9
Work Accident	12	7.3
Liver Disease	9	5.5
Heart disease	8	4.8
Psychiatric Disease	8	4.8
Pregnancy Monitoring	7	4.2
Diabetes	7	4.2
Oncological Disease	7	4.2
Other	7	4.2
Vaccination	6	3.6
Neurological Disease	6	3.6
Personal accident	6	3.6
Childbirth	4	2.4
Children's health	4	2.4
Chemotherapy	3	1.8
Radiotherapy	2	1.2
Dentistry	0	0
Car Accident	0	0

Each participant could choose more than one option (hence the sum being > 165).
